# Transcriptional and Post-Translational Targeting of Myocyte Stress Protein 1 (MS1) by the JNK Pathway in Cardiac Myocytes

**DOI:** 10.5334/1750-2187-12-3

**Published:** 2017-12-08

**Authors:** Joanna M. Hay, Eva S. Jordan, Gareth J. Browne, Andrew R. Bottrill, Sally A. Prigent, Martin Dickens

**Affiliations:** 1 Department of Molecular & Cell Biology, Henry Wellcome Building, University of Leicester, Lancaster Road, Leicester, LE1 7RH, UK; 2 Proteomics Facility, Protein and Nucleic Acid Chemistry Laboratory, Hodgkin Building, University of Leicester, Lancaster Road, Leicester, LE1 7RH, UK; 3 Human Physiology, College of Health, Massey University at Albany, North Shore, Auckland, 0630, NZ

**Keywords:** MS1/STARS, JNK, Metabolic Stress

## Abstract

Myocyte Stress Protein 1 (MS1) is a muscle-specific, stress-responsive, regulator of gene expression. It was originally identified in embryonic mouse heart which showed increased expression in a rat model of left ventricular hypertrophy. To determine if MS1 was responsive to other stresses relevant to cardiac myocyte function, we tested if it could be induced by the metabolic stresses associated with ischaemia/reperfusion injury in cardiac myocytes. We found that metabolic stress increased MS1 expression, both at the mRNA and protein level, concurrent with activation of the c-Jun N-terminal Kinase (JNK) signalling pathway. MS1 induction by metabolic stress was blocked by both the transcription inhibitor actinomycin D and a JNK inhibitor, suggesting that activation of the JNK pathway during metabolic stress in cardiac myocytes leads to transcriptional induction of MS1. MS1 was also found to be an efficient JNK substrate *in vitro*, with a major JNK phosphorylation site identified at Thr-62. In addition, MS1 was found to co-precipitate with JNK, and inspection of the amino acid sequence upstream of the phosphorylation site, at Thr-62, revealed a putative Mitogen-Activated Protein Kinase (MAPK) binding site. Taken together, these data identify MS1 as a likely transcriptional and post-translational target for the JNK pathway in cardiac myocytes subjected to metabolic stress.

## Introduction

Myocyte stress protein 1 (MS1) is a myofibrillar protein involved in the regulation of gene expression in both cardiac and skeletal muscle. It is also known as striated muscle activator of Rho Signalling and Serum Response Factor dependent transcription (STARS)[1] or Actin-binding Rho-activating protein (ABRA)[2]. MS1 was originally identified in the developing mouse heart and as a gene showing increased expression during pressure-induced left ventricular hypertrophy, suggesting its involvement in cardiac remodelling and the hypertrophic response [1,3,4].

More recent evidence suggests that MS1 likely plays a key role in regulating many aspects of myocyte cell physiology during development, in regeneration after injury and also in the adaptive responses to metabolic and mechanical stresses experienced by both cardiac and skeletal muscle [5,6,7]. MS1 associates with actin at the myofibril via two actin binding domains in the C-terminus of the protein [1,8]. MS1 is concentrated at the I-band and Z-disk of the sarcomere where it may act as a mechanosensor, transducing signals in response to mechanical loading of the muscle during contraction or when the muscle is stretched. The exact mechanisms are not completely clear, but MS1 interacts with a Rho A GTPase and stimulates the polymerisation of G-actin to F-actin, causing the depletion of cytosolic G-actin. G-actin thereby dissociates from the Myocardin Related Transcription Factor-A (MRTF-A), allowing it to translocate to the nucleus, where, in collaboration with Serum Response Factor (SRF) acting at the Serum Response Element (SRE), it switches on the hypertrophic gene programme involved in muscle cell proliferation, growth and repair [1,4,9,10].

Many of the adaptive responses of cardiac myocytes to the pathophysiological processes associated with cardiovascular disease involve activation of Mitogen-Activated Protein Kinase (MAPK) pathways [11,12]. These pathways mediate changes in gene expression by targeting transcription factors, including those that activate the SRE [13,14,15,16]. The stress-activated MAPKs, c-Jun N-terminal Kinase (JNK) and p38-MAPK, are activated strongly by cardiac ischaemia/reperfusion injury [15,16,17,18]. The activity of p38 is increased by ischaemia and maintained during reperfusion, in contrast to JNK, which is activated by redox stress when the heart is reperfused after ischaemia [12,17,19,20,21,22]. Both the JNK and p38 pathway are involved in mediating diverse adaptive responses in myocytes, including protection from redox stresses, ischaemic preconditioning and the induction of muscle hypertrophy [11,17]. MS1 induction by mechanical loading activates downstream stress-responsive pathways converging on ternary complex factors (TCF)/SRF [1,9], which are a known target of stress-activated MAPKs such as JNK [13,14]. In addition, JNK is involved in the physiological response to exercise in skeletal muscle, which also involves MS1 [23,24,25,26,27]. These observations suggest that MS1 is likely to be regulated by multiple stress-responsive pathways in myocytes and that MS1 and JNK signalling pathways may converge at some point. The aim of our study was, therefore, to determine if MS1 was responsive to other cellular stresses relevant to cardiac myocytes and investigate possible links between MS1 and JNK activation. To do this we tested (i) if MS1 could be induced by the same metabolic/redox stresses associated with ischaemia/reperfusion injury, which also activate the JNK pathway, and (ii) if MS1 was a direct target for the JNK pathway.

## Materials and Methods

### Antibodies and reagents

Anti-phospho JNK (Cat. No. 9251S), anti-JNK (Cat. No. 9252S) and phospho-c-Jun (Cat. No. 9146) were purchased from Cell Signalling Technologies (Beverley, MA, USA). Anti-a-tubulin (Cat. No. T5168) and anti-Flag M2 (Cat. No. F-3165) were purchased from Sigma (Poole, U.K.). Anti-HA (Cat. No. Sc805) was from Santa Cruz Biotechnology (Santa Cruz, California, USA). A rabbit polyclonal antiserum against full-length rat MS1 (1–375) was produced at Cambridge Research Bioscience (Cambridge, UK). Anti-rabbit and anti-mouse horseradish peroxidase (HRP) conjugates were purchased from GE Healthcare Life Sciences (Amersham, UK). Dulbecco’s Modified Eagle Medium (DMEM), penicillin, streptomycin, collagenase, heat-inactivated fetal calf serum (FCS), new-born calf serum (NCS) and optiMEM were purchased from Invitrogen (Paisley, UK). Pancreatin (Cat. No. P3292) and gelatin (Cat. No. G2500) were purchased from Sigma (Poole, UK). The inhibitors SP600125 and actinomycin D were from CN Biosciences Inc (San Diego, CA, USA).

### Plasmids and proteins

pETM-11-MS1 expression plasmids and pcDNA3-HA-JNK1 were generous gifts from Dr. Mark Pfuhl (Kings College, London) and Dr. Raj Patel (University of Leicester) respectively. The pFlag-CMV2-MS1 expression vector was constructed by cloning the full-length rat MS1 cDNA between the HindIII and EcoRI sites of the pFlag-CMV2 polylinker (Sigma). A threonine to alanine mutation at residue 62 (T62A) of MS1 was made using the Quikchange site directed mutagenesis kit (Stratagene, La Jolla, CA, USA). Plasmid vectors were maintained in *E. coli* DH5α and plasmid DNA for transfection prepared using the QIAprep Maxiprep Kit according to the manufacturer’s instructions (Qiagen, Manchester, UK).

Glutathione S-transferase (GST) and GST-Jun (1–79) were expressed in *E.coli* BL21-DE3 using pGEX vectors and purified by glutathione agarose affinity chromatography as described previously [28]. Full-length rat MS1 (residues 1–375), an N-terminal MS1 fragment (1–118) and a T62A mutant cloned in pETM-11 were expressed as His-tagged proteins in *E.coli* BL21-DE3. Proteins were purified by metal-ion affinity chromatography on nickel-chelating sepharose 6 Fast Flow (GE Healthcare Life Sciences). After cleavage of the His-tag, proteins were further purified by gel filtration on a Superdex 75 HiLoad 16/60 column using an Äkta Purifier 9 and Unicorn software (GE Healthcare Life Sciences) as described previously [8,28].

### Isolation of neonatal cardiac myocyte cells

Neonatal cardiac myocytes were isolated from 1–3 day old rats. Myocytes were prepared from the excised heart as described previously [29,30]. Fibroblasts were depleted by pre-plating the cells for 1 h at 37°C on uncoated 10 cm dishes in maintenance medium (DMEM/medium 199 (4:1 (v/v)), 100 U/ml penicillin and streptomycin, 10% (v/v) horse serum, 5% (v/v) FCS). Unbound myocytes were removed and plated at a final density of 350 cells/mm^2^ on 60 mm gelatin-coated dishes. After 20 hours the medium was replaced with fresh maintenance medium. Each heart yielded approximately 1 × 10^6^ myocytes. The work in this study was conducted in accordance with the Animals (Scientific Procedures) Act 1986 under Home Office licence. Ethical review, project approval and animal care were provided by the University of Leicester Division of Biomedical Services.

### Cell Culture and Transfection

H9c2 rat cardiac myoblasts [31] and HEK-293 cells [32] were kind gifts from Dr. I. Eperon and Dr. T. Herbert respectively (University of Leicester). Both cell lines were routinely cultured in DMEM containing 10% FCS, 100 U/ml penicillin & streptomycin at 37°C in an atmosphere of 5% CO_2_. For transfections, 1 × 10^5^ HEK-293 cells were seeded onto 60 mm cell culture dishes in DMEM (10% FCS). Cells were transfected using TurboFect Transfection Reagent (Life Technologies) at a lipid: DNA ratio of 3:1 (µl/µg) according to the manufacturer’s instructions.

### Metabolic stress and recovery

Sixteen hours prior to the experiment cells were transferred to low-serum DMEM (2% FCS). Metabolic stress was induced by transferring cells to a modified Krebs buffer (4 mM HEPES, 5.6 mM glucose, 137 mM NaCl, 16 mM KCl, 0.49 mM MgCl_2_, 0.9 mM CaCl_2_, 2% FCS, pH 6.5) containing metabolic inhibitors (10 mM 2-deoxyglucose, 1 mM sodium dithionite, 20 mM sodium lactate) [20,30,33,34,35]. Cells were allowed to recover from metabolic stress by returning them to low-serum DMEM (2% FCS), whilst control cells were maintained in low-serum DMEM throughout.

### Northern Blotting

Total RNA was isolated using Tri-reagent (Sigma), from neonatal cardiac myocytes and H9c2 rat cardiac myoblasts plated on 60 mm dishes. Equal amounts of total RNA (10 µg) were separated on denaturing 1% (w/v) agarose/formaldehyde/MOPS gels. After electrophoresis gels were rinsed in water, soaked in 0.05 M NaOH for 20 minutes, and then in 20 × SSC (3 M NaCl, 0.3 M Na citrate pH 7.0) for 30 min. RNA was transferred by capillary action to nylon membranes and fixed by baking at 80°C for 90 minutes prior to pre-hybridisation at 65°C in Church-Gilbert solution (100 mM Na_2_HPO_4_, 70 mM NaH_2_PO_4_, 7% (w/v) SDS, 10 µg/ml salmon sperm DNA) for 90 min. Membranes were hybridised for 16 h at 65°C with heat denatured [α-^32^P] dCTP-labelled probes generated by random priming of cDNAs (Prime-IT II kit, Stratagene). Membranes were then washed twice for 20 minutes with wash solution I (1 × SSC, 0.1% (w/v) SDS), and 3 times for 20 minutes with wash solution II (0.2 × SSC, 0.1% (w/v) SDS). Levels of mRNA were quantified by PhosporImager analysis of the dried membranes (GE Healthcare Life Sciences) [34].

### RT-PCR Analysis

Neonatal cardiac myocytes and H9c2 rat cardiac myoblasts plated on 60mm dishes were extracted using Tri-reagent (Sigma), total RNA isolated and cDNA prepared by reverse transcription from 1 µg of RNA using oligo (dT) primers (RT kit, Promega, Southampton, UK). Sequences corresponding to MS1, actin and c-fos were amplified using the polymerase chain reaction (PCR) with cDNA from 0.2 µg of reverse transcribed RNA as template. MS1 was amplified for 22 cycles (denaturation: 90 s at 95°C, annealing: 45s at 59°C, extension: 45 s at 72°C) using primers: 5’ – TAG ACA CAG AGG ACA GTG GCT ACG – 3’ / 5’ – CCG AAA GTA ACC TGG ATC TTG C – 3’. VEGF-A was amplified for 35 cycles (denaturation: 90s at 95°C, annealing: 120 s at 57°C, extension: 120 s at 72°C) using primers: 5’ – TGC TGT ACC TCC ACC ATG CCA – 3’ / 5’ – CTG CAA GTA CGT T CG TTT AAC – 3’ as described previously [34]. Actin and *c-fos* were amplified for 16 cycles (denaturation: 90 s at 95°C, annealing: 120 s at 57°C, extension: 120 s at 72°C) using primers: 5’ – CAA CCG TGA AAA GAT GAC – 3’ / 5’ – CCA GAC AGC ACT GTG TT – 3’ for actin and 5’ – CAC CAG CCC AGA CCT GCA GTG GCT – 3’ / 5’ – GAG GCA GGG TGA AGG CCT CCT CAG – 3’ for *c-fos*. Cycle numbers were in the range where log (RT-PCR signal) and cycle number were linearly related. No PCR products were detected when reverse transcriptase was omitted. The identities of all PCR products were confirmed by automated DNA sequencing (Applied Biosystems). Amplicons were detected by Southern blotting using [α-^32^P] dCTP-labelled probes generated by random priming of cDNAs using the Prime-IT II kit (Stratagene). Bands were quantified by PhosphorImager analysis and statistical significance determined by a global one-way analysis of variance (ANOVA) at a significance level of 95% (α = 0.05) [34].

### Western Blotting

H9c2 cardiac myoblasts were washed once with ice-cold phosphate-buffered saline (PBS) and lysed by scraping into ice-cold lysis buffer (20 mM HEPES, 137 mM NaCl, 25 mM β-glycerophosphate, 2 mM NaPPi, 2 mM EDTA, 10% glycerol, 10 µl/ml protease inhibitor cocktail (Sigma), 0.5 mM DTT, 1 mM Na_3_VO_4_, 1% Triton X-100; pH 7.4). Cell lysates (100 µg), were separated on 10% polyacrylamide/tricine gels [36], proteins transferred to Hybond-ECL membranes (GE Healthcare Life Sciences) and western-blotted using primary antibodies at the following dilutions: anti-JNK, anti-Phospho-JNK and anti-c-Jun (1:1,000); anti-α-tubulin (1:10,000). Primary antibody was diluted in 5% (w/v) milk or 3% (w/v) BSA in tris-buffered saline, 0.1% (v/v) Tween-20 (TBST). Washes were in TBST. Blots were visualised using horseradish peroxidase coupled secondary antibodies diluted (1:10,000) in 5% milk/TBST and with Enhanced ChemiLuminescence detection (GE Healthcare Life Sciences).

### Immunoprecipitation and immune-complex kinase assay

Transfected HEK-293 cells were washed once in ice-cold PBS and lysed in ice-cold lysis buffer. Cell lysates were clarified by centrifugation at 20,000 × g for 15 min at 4°C. Cell lysates (approx. 500 µg total protein) were immunoprecipitated by tumbling for 3 h at 4°C with 5 mg Protein-A Sepharose (Sigma) and 5 µl of anti-Flag, anti-HA or control antibody in a final volume of 500 µl. Immune complexes were isolated by brief low-speed centrifugation at 4°C and washed three times with 1-ml of ice-cold lysis buffer. Immunoprecipitates were either resuspended in sample buffer before SDS-PAGE and western blotting or used for JNK immune-complex kinase assays. For kinase assays the immunoprecipitates were washed once with 1ml kinase assay buffer (25 mM HEPES, pH 7.4, 25 mM β-glycerol phosphate, 25 mM MgCl_2_, 0.5 mM Na_3_VO_4_, 0.5 mM DTT) and then resuspended in the same buffer to a final volume of 50 µl containing 50 µM [β-^32^P] ATP (2000 cpm/pmol) and 2 µg of purified protein substrate: either GST, GST-Jun (1–79) or MS1 (1–118). Kinase assays were incubated for 30 min at 30°C and terminated by the addition of SDS-PAGE sample buffer prior to electrophoresis on 10% SDS-PAGE gels. Incorporation of ^32^P into substrates was quantified by PhosporImager analysis of the dried gels. [22,28].

### MS1 Phosphorylation site analysis by LC-MS/MS

Bands corresponding to phosphorylated MS1 were identified by PhosphorImager analysis of dried gels. Bands of interest were excised from the gel, reduced with DTT, alkylated with iodoacetamide and subjected to in-gel tryptic digestion prior to analysis by LC-MS/MS [37]. Using an RSLCnano HPLC system (Dionex, Hemel Hempstead, UK), tryptic peptides were loaded onto a reverse-phase trap column (0.3 mm i.d. × 1 mm) containing 5 μm C18 300 Å Acclaim PepMap media (Dionex) at 37°C in 0.1% formic acid / 0.05% trifluoroacetic acid / 2% acetonitrile. Peptides were eluted at a flow rate of 0.3 µl/min and onto a reverse-phase PicoFrit capillary column (75 μm i.d. × 400mm) containing Symmetry C18 100 Å media (Waters, Elstree, UK). Eluted peptides were analysed using an LTQ-Orbitrap-Velos mass spectrometer set to acquire a 1 microscan FTMS scan event at 60000 resolution over the m/z range 350–1250 Da in positive ion mode (Thermo Scientific). Mass spectrometry data were analysed using Proteome Discoverer (Thermo Scientific), Mascot2 (Matrix Science Ltd.), Scaffold Q+S4 (Proteome Software) and the UniProtKB-Swissprot3 database.

## Results

### Induction of MS1 mRNA during recovery from metabolic stress in cardiac myocytes

**Figure 1:**
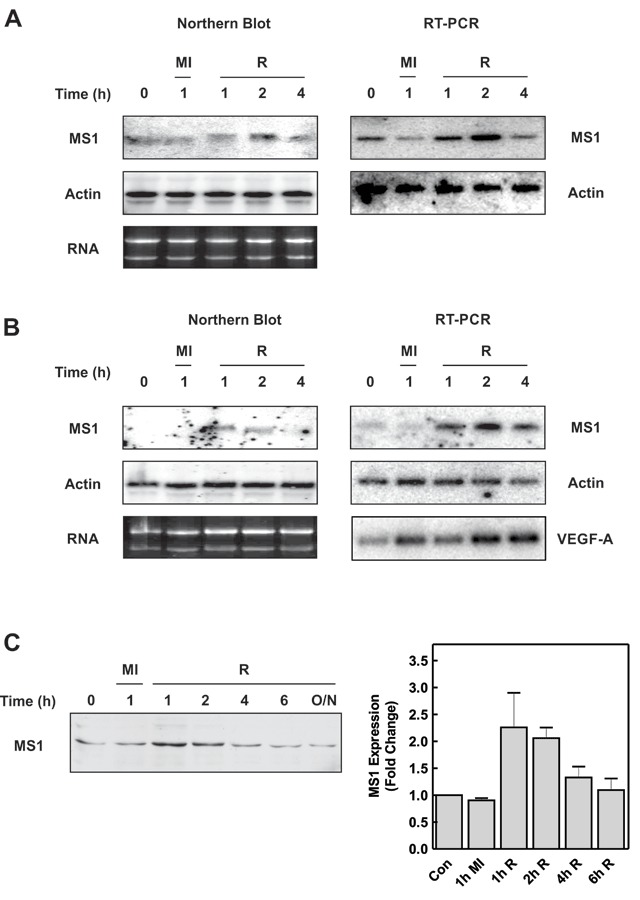
**MS1 expression is increased during metabolic stress in cardiac myocytes.** Neonatal cardiac myocytes **(A)** or H9c2 cells **(B)** were subjected to metabolic inhibition (MI) or allowed to recover (R) following 1h of metabolic inhibition for the times indicated. Northern blots for MS1 and β-Actin mRNA are presented on the left-hand side with MS1, VEGF-A and β-Actin mRNA determined by RT-PCR on the right. Ethidium bromide-stained gels showing 28S and 18S RNA levels are also shown. **(C)** Cell extracts from H9c2 cells subjected to metabolic inhibition (MI) and recovery (R) were blotted with antibodies to MS1. A representative blot and a graph indicating quantification of the data are shown (mean ± SEM [n = 3]).

To determine if the cellular stresses associated with ischaemia-reperfusion injury affected the level of MS1 expression in cardiac myocytes, MS1 mRNA levels in both neonatal cardiac myocytes and H9c2 cardiac myoblasts were determined by northern blotting and RT-PCR (Fig. 1A & 1B) following treatment with metabolic inhibitors and recovery. Equivalent results were obtained in both cell types using both techniques. In untreated cells, MS1 mRNA levels were very low and this was not changed by exposure to metabolic inhibitors for 1 h. However, after 1 h of the recovery period there was a clear increase in MS1 mRNA levels which reached a maximum at 2 h (Fig. 1A & 1B). As expected, metabolic stress also increased the mRNA level of Vascular Endothelial Growth Factor-A (VEGF-A), indicating that the cells were hypoxic (Fig. 1B) [34]. Although increased MS1 mRNA expression was readily detected in cells recovering from metabolic stress, it was difficult to detect expression of MS1 in control cells, either H9c2 or neonatal cardiac myocytes, by northern blotting without using large quantities of RNA. Consequently, we chose to use RT-PCR to measure changes in MS1 mRNA levels in subsequent experiments. Since the results were similar in both H9c2 cells and primary neonatal cardiac myocytes the H9c2 cell line was used for the remainder of the experiments.

Increased levels of MS1 mRNA should give rise to increased levels of the MS1 protein. To confirm this, we measured MS1 protein levels by immunoblotting cell lysates from H9c2 subjected to metabolic stress (Fig. 1C). As expected, MS1 levels were increased during the recovery period mirroring the increase in MS1 mRNA (Fig. 1B).

### Increased MS1 mRNA is due to increased transcription

**Figure 2:**
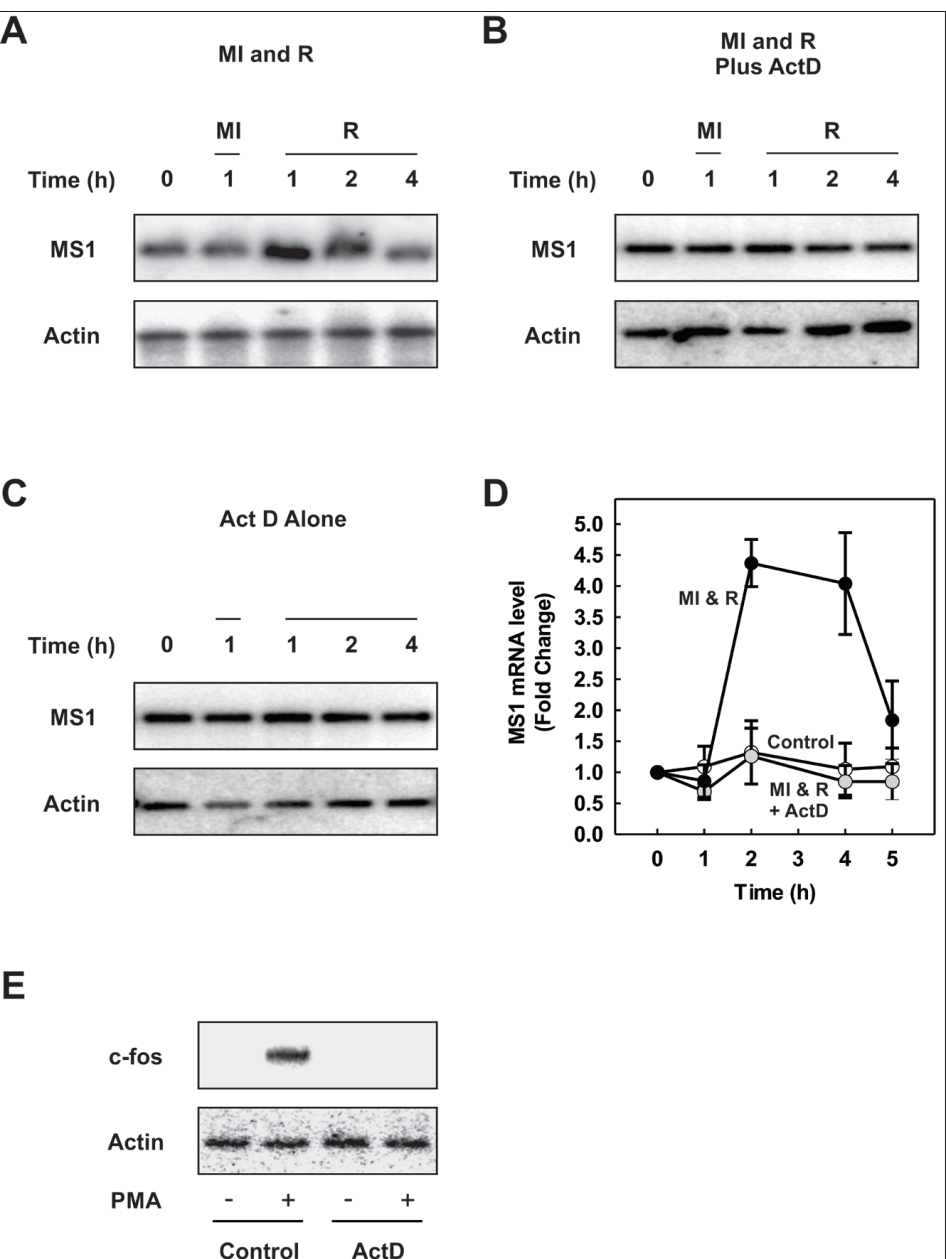
**Increased levels of MS1 are due to transcription. (A)** H9c2 cells were subjected to metabolic inhibition (MI) or allowed to recover (R) following 1h of metabolic inhibition for the times indicated. **(B)** H9c2 cells were preincubated with 5 µg/ml actinomycin-D for 1h then subjected to metabolic inhibition (MI) and recovery (R) in the continued presence of actinomycin-D, for the times indicated. **(C)** H9c2 cells were incubated in actinomycin-D for the times as indicated. MS1 and β-actin mRNA levels were determined by RT-PCR. **(D)** MS1 mRNA levels normalised to β-actin are presented graphically (mean ± SEM [n = 3]). **(E)** H9c2 cells were preincubated with or without 5 µg/ml actinomycin-D for 1h prior to treatment with 1 µM PMA for 30 minutes as indicated. Total RNA was isolated and c-fos and β-actin gene expression determined by RT-PCR.

To investigate the nature of the induction of MS1 mRNA in H9c2 cells we used the transcription inhibitor actinomycin-D. H9c2 cells were pre-incubated with or without actinomycin D for 1 h prior to the start of the experiment, treated with metabolic inhibitors and allowed to recover as indicated in Figure 2. H9c2 cells incubated in the absence of actinomycin-D showed the expected induction of MS1 mRNA during the recovery period after metabolic stress (Fig. 2A). However, MS1 induction by metabolic stress was abolished in the presence of actinomycin-D showing that increased transcription may be responsible for the increases in MS1 mRNA seen (Fig. 2B). In H9c2 cells incubated in the presence of actinomycin-D but not exposed to metabolic stress, basal levels of MS1 mRNA were maintained throughout the time course of the experiment, showing that MS1 mRNA was inherently stable (Fig. 2C). This suggests that changes in mRNA levels in response to metabolic stress are unlikely to be due to changes in mRNA stability. The results are presented graphically in Fig. 2D. The efficacy of actinomycin-D as an inhibitor of transcription in H9c2 cells was confirmed by its ability to inhibit the transcriptional induction of *c-fos* in response to PMA (Fig. 2E).

### Role of the JNK pathway in regulating MS1 induction

**Figure 3:**
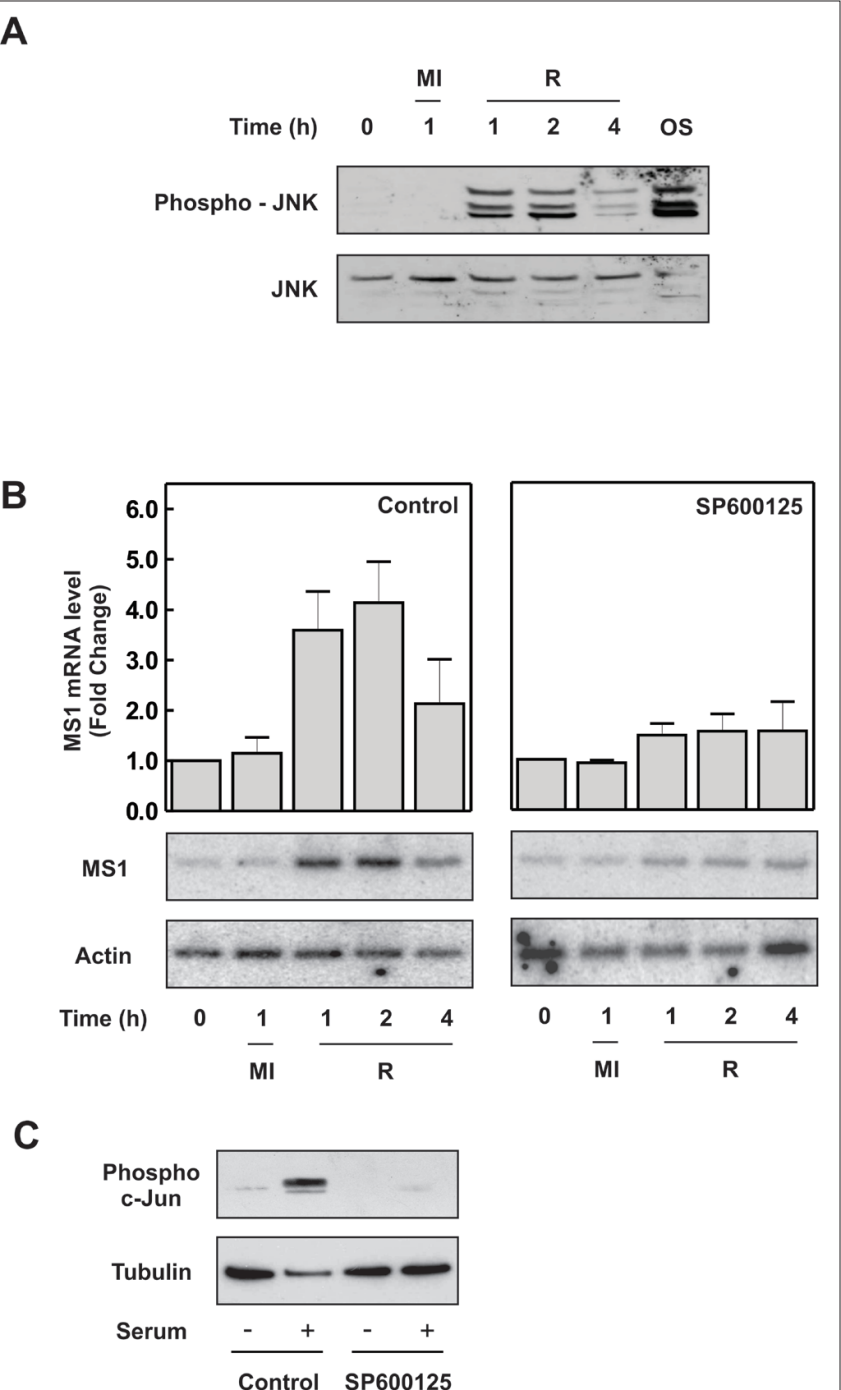
**JNK activation is required for increased MS1 transcription during metabolic stress. (A)** H9c2 cells were subjected to metabolic inhibition (MI) or allowed to recover (R) following 1h of metabolic inhibition for the times indicated. As a positive control, JNK was activated via osmotic shock, by exposure of the cells to 0.5 M sorbitol for 30 minutes (OS). Cell extracts were separated by SDS-PAGE and subjected to western blotting with antibodies reactive to phosphorylated (active) and total JNK as indicated. **(B)** H9c2 cells were preincubated without or with 25 µM SP600125, for 1 h and then subjected to metabolic inhibition (MI) or allowed to recover (R) following 1h of metabolic inhibition in the continued presence of the JNK inhibitor. MS1 and β-actin mRNA levels were determined by RT-PCR and MS1 normalised against β-actin. Results are the mean ± SEM (n = 3). **(C)** In control experiments, serum-starved H9c2 cells were preincubated with 25 µM SP600125 for 1 h prior to incubation with 10% serum, to activate JNK. Cell lysates were then blotted for phospho-c-Jun and tubulin.

The JNK signalling pathway is activated by a wide variety of stimuli, particularly cellular stresses, and plays a major role in the regulation of gene expression by the phosphorylation of transcription factors. To determine if JNK was involved in the transcriptional induction of MS1 by metabolic stress we subjected H9c2 cells to metabolic inhibition and allowed them to recover. The activity of JNK was then assayed by western blotting of the cell extracts with antibodies reactive to the phosphorylated, active form of JNK (Fig. 3A). In unstimulated cells, and in cells under-going metabolic inhibition, JNK activity was undetectable. However, JNK was clearly activated in cells recovering from metabolic inhibition and also, in a control experiment, where cells were exposed to osmotic shock by treatment with 0.5 M sorbitol (OS). JNK activation occurred with a similar time course to the induction of MS1, suggesting that JNK signalling could be involved in the transcriptional induction of MS1 by metabolic stress (Fig. 3A). To test this, we examined the effect of JNK inhibition on the ability of metabolic stress to induce MS1. H9c2 cells subjected to metabolic inhibition and allowed to recover showed the expected induction of MS1 with relative increases of MS1 mRNA levels of 3.6 ± 0.8 and 4.1 ± 0.8-fold after 1 and 2 hours’ recovery respectively (Fig. 3B). However, when the experiment was repeated in the presence of the JNK inhibitor, SP600125, the induction of MS1 by metabolic stress was attenuated significantly, decreasing to 1.5 ± 0.2 and 1.6 ± 0.3-fold at 1 and 2 hours respectively (Fig. 3B). The ability of SP600125 to inhibit JNK activation was confirmed by the inhibition of c-Jun phosphorylation in response to serum stimulation (Fig. 3C). This result strongly implicates activation of the JNK pathway in the transcriptional induction of MS1 by metabolic stress.

### MS1 protein is also a target for JNK

**Figure 4:**
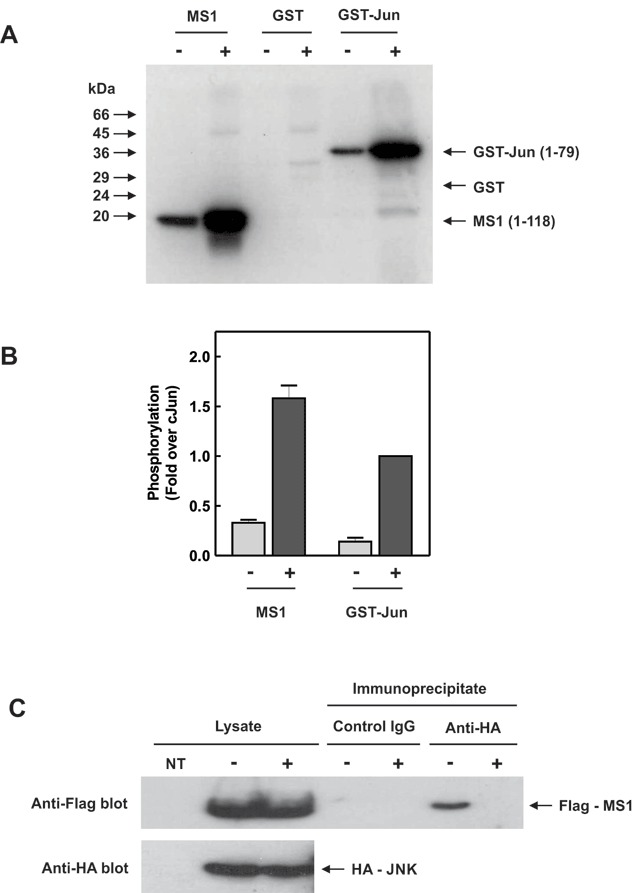
**MS1 is a JNK target. (A)** HEK cells, transfected with pcDNA3-HA-JNK and expressing HA-tagged JNK, were incubated with (+) or without (–) 0.5 M sorbitol for 30 minutes to activate the JNK pathway. HA-JNK was immunoprecipitated from the cell lysates and incubated with [γ-^32^P] ATP along with a purified N-terminal fragment of MS1 (1–118), GST or GST-Jun (1–79) in an *in vitro* immune-complex kinase assay. ^32^P-incorporation into substrates was determined by SDS-PAGE and PhosphorImager analysis. **(B)** Quantification was performed using ImageQuant software and the relative phosphorylation of MS1 compared to GST-Jun is shown (mean ± SEM [n = 3]). **(C)** HEK cells were co-transfected with pcDNA3-HA-JNK and pFlag-CMV2-MS1 to express both HA-tagged JNK and Flag-tagged MS1. The cells were treated with 0.5 M sorbitol for 30 min (+) or left untreated (–) and cell lysates immunoprecipitated with anti-HA antibody or control IgG. Cell lysates and immunoprecipitates were separated by SDS-PAGE and blotted with anti-Flag and anti-HA antibodies. Non-transfected cell lysates were run as a negative control (NT).

**Figure 5:**
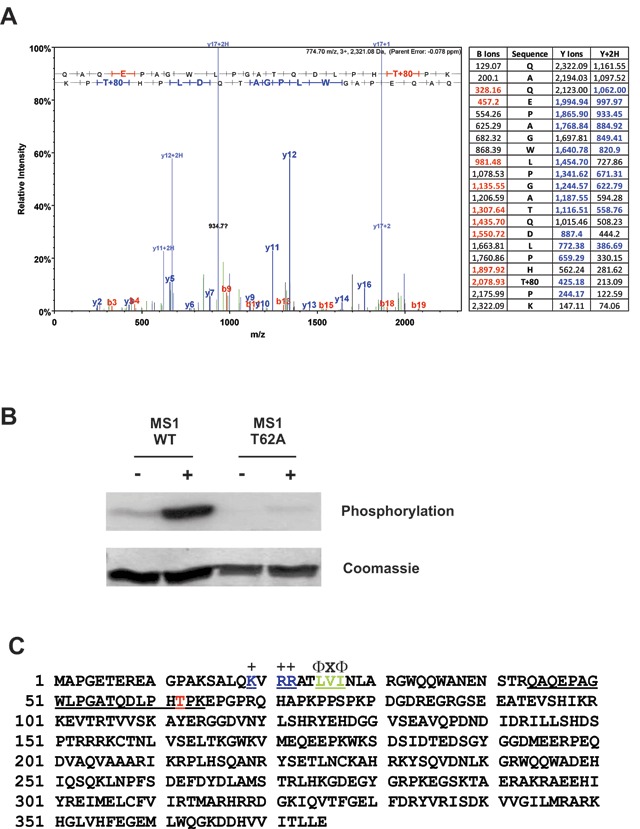
**JNK phosphorylates MS1 in vitro at Threonine-62. (A)** The N-terminal fragment of MS1 (1–118) was phosphorylated by JNK *in vitro* as described in Fig. 4A but using unlabelled ATP. The Coomassie-stained band corresponding to the MS1 substrate was excised from the gel and subjected to phosphorylation site analysis by tryptic digestion followed by LC-MS/MS. The LC-MS/MS trace and fragmentation table showing the detected b-ions (red) and y-ions (blue) are presented. **(B)** Active (+) or inactive (–) HA-JNK immunoprecipitated from HEK cells as described in Fig. 4A was incubated with [γ-^32^P] ATP and either the wild-type MS1 N-terminal fragment (1–118) or the same protein with a T62A mutation. ^32^P-incorporation was determined by SDS-PAGE and PhosphorImager analysis. **(C)** MS1 amino acid sequence showing the tryptic peptide (residues 44–64, underlined) containing Thr-62 (red) and the location of a putative MAPK docking site (residues 19–27, underlined with consensus sequence above).

Since the transcriptional induction of MS1 by metabolic stresses appeared to require JNK activation, we were interested to determine if the JNK kinase may play a more direct role in MS1 function. Inspection of the MS1 protein sequence (Fig. 5C) revealed several potential MAPK phosphorylation sites in the N-terminus of the protein, suggesting that MS1 might be a JNK substrate. To test this, we investigated whether an N-terminal fragment of MS1 (residues 1-118) could serve as a JNK substrate in an *in vitro* immune-complex kinase assay. Epitope-tagged JNK, that was inactive or activated by osmotic shock (0.5 M sorbitol), was immunoprecipitated from transfected HEK-293 cells and used in a kinase assay with an N-terminal fragment of MS1, GST or GST-Jun (1–79) as substrates (Fig. 4A, 4B). The N-terminus of MS1 proved to be an excellent substrate for JNK *in vitro*, comparable to that of GST-Jun, the long-established substrate for JNK, with a level of phosphorylation 1.58 ± 0.13-fold over GST-Jun phosphorylation (Fig. 4B). In control assays GST was not phosphorylated by JNK (Fig. 4A).

JNK recognises its main substrate, c-Jun, by binding to a region in its N-terminus containing the phosphorylation sites at Ser-63 and Thr-73 [38]. To determine if MS1 interacts with JNK in a similar way, we tested whether JNK and MS1 could co-immunoprecipitate from HEK cells expressing HA-tagged JNK and Flag-tagged MS1. Both HA-JNK and Flag-MS1 were efficiently expressed in HEK cells, being readily detected in the cell lysate with no signal detected in non-transfected cells (Fig. 4C). Control IgG was unable to precipitate Flag-tagged MS1 from either unstimulated or stimulated HEK cell lysates. In contrast, anti-HA immunoprecipitates from unstimulated, but not stimulated cells were found to contain Flag-tagged MS1 suggesting that MS1 physically associates with inactive, but not active JNK. The presence of an HA-tagged JNK in the immunoprecipitates was obscured by the heavy chain of the precipitating antibodies and is not shown.

### JNK phosphorylates MS1 at Thr-62

To identify the sites on MS1 phosphorylated by JNK, the N-terminal fragment of MS1 (1–118) was phosphorylated *in vitro* and subjected to tryptic digestion followed by Liquid Chromatography-tandem Mass Spectrometry (LC-MS/MS) at the University of Leicester Proteomics Facility. Analysis of the spectrum and fragmentation pattern indicated phosphorylation at Thr-62 (Fig. 5A). To confirm the presence of a JNK phosphorylation site at Thr-62 of MS1 we examined the *in vitro* phosphorylation of a Thr-62-Ala mutant by JNK compared to the wild-type protein (Fig. 5B). JNK efficiently phosphorylated the wild-type protein but phosphorylation of the mutated substrate was almost completely abolished, confirming Thr-62 as a major JNK phosphorylation site *in vitro* (Fig. 5C).

## Discussion

Skeletal and cardiac muscle are plastic tissues that adapt to both the physiological stresses associated with normal contractile function and the pathophysiological stresses associated with disease. Such adaptive responses involve integration of signals arising from many sources, including mechanical loading of the muscle, metabolic perturbations and associated redox stress. Stress-activated signalling pathways couple these signals to downstream responses such as changes in gene expression [11,16,17,39]. The recent literature on MS1 [5,6,10,27,40,41,42] and our previous work on stress-activated signalling pathways in cardiac myocytes [22,33,34] suggested that MS1 might be induced by metabolic stress in cardiac myocytes and that there may be a link between the JNK signalling pathway and MS1.

### MS1 induction by metabolic stress

MS1 was originally identified as a gene induced by pressure overload in a rat model of left ventricular hypertrophy [1,3]. Since hypertrophy of surviving cardiac myocytes is one of the adaptive responses to ischaemia/reperfusion injury we decided to test if MS1 might be induced by the metabolic and redox stresses associated with cardiac ischaemia/reperfusion. In both primary rat neonatal cardiac myocytes and the rat H9c2 cardiomyoblast cell line we found that metabolic stress led to induction of MS1 at the mRNA and protein level (Fig. 1). The transcriptional inhibitor, Actinomycin D completely blocked this effect showing it to be due to stimulation of transcription (Fig. 2). It is currently unknown if MS1 is upregulated during cardiac ischaemia/reperfusion *in vivo* or in the isolated, perfused heart, and further work will be required to determine this. However, MS1 is involved in the physiological adaptations that occur in skeletal muscle during exercise. Both endurance and resistance training in skeletal muscle bring about an increase MS1 expression via intracellular signals generated in response to the mechanical stresses of contraction, suggesting that MS1 expression may be regulated by a wide variety of cellular stresses *in vivo* [26,27,43,44,45].

### Transcriptional induction of MS1 by the JNK pathway

The JNK pathway is a major stress-responsive pathway activated by ischaemia/reperfusion injury in the heart [11,12,17]. Our previous work showed that the metabolic stresses associated with ischaemia/reperfusion injury activate the JNK pathway in H9c2 cells [34]. Since JNK is a key regulator of transcription we decided to test if JNK activity were required for MS1 induction by metabolic stress in H9c2 cells.

The kinetics of JNK activation during metabolic stress were strikingly similar to those of MS1 induction, with both occurring only upon recovery from metabolic inhibition (Fig. 3).

Pharmacological inhibition of JNK was found to completely abolish the transcriptional induction of MS1 (Fig. 3B), suggesting that the MS1 promoter may be a downstream target for the JNK signalling pathway. Generation of cells with specific knockdown of JNK isoforms would support the involvement of JNK and also address which isoforms are important for the effect on MS1 transcription.

The MS1 promoter has been characterised in some detail with binding sites for several transcription factors having been identified and some of these may be JNK targets [10,40,41,46]. Of particular interest is an SRF binding site at an SRE located 305 base pairs upstream of the transcription start site [40]. SRF is known to interact with a family of TCFs which are directly phosphorylated by JNK and other MAPKs, thus providing a possible mechanism for JNK mediated regulation of the MS1 promoter [13,14,47]. MS1 promoter analysis has also revealed two functional E-Box sequences (CANNTG) located at –1515/–1509 and –253/–247 [46]. These bind the myogenic regulatory factor, MyoD, and likely contribute to the muscle-specific gene expression of MS1. Since MyoD is known to interact with the transcription factor AP-1, components of which are JNK substrates, JNK activity might influence MS1 expression via AP-1 and MyoD [48,49,50,51]. Other potential, although indirect, JNK targets in the MS1 promoter include the transcription factors MEF2 (binding site at –135/–125) and GATA4, with multiple potential binding sites in the MS1 promoter. Expression levels of both of these transcription factors may be regulated by JNK [52]. Further analysis of the MS1 promoter using reporter constructs will be required to determine which regions of the promoter mediate the effect of JNK on MS1 transcription.

### MS1 is a JNK substrate in vitro

The involvement of both JNK and MS1 in mediating hypertrophic transcriptional responses led us to investigate the possibility of more direct links between JNK activation and MS1. Inspection of the amino acid sequence of MS1 showed potential MAPK phosphorylation sites (S/T–P), close to the N-terminus of the protein. We therefore tested a purified N-terminal fragment of MS1 (residues 1–118) as an *in vitro* substrate for JNK. This protein was an excellent substrate for JNK, appearing comparable with its canonical substrate c-Jun, with a major phosphorylation site identified at Thr-62 (Figs. 4A, 4B and 5).

The substrate selectivity of MAPKs usually involves interaction with their substrates via a MAPK docking motif [16,53]. JNK interacts with its substrate c-Jun via such a motif that lies upstream of the main JNK phosphorylation sites at Ser/Thr 63 and 73 [38]. Phosphorylation at these sites plays a critical role in JNK-mediated transcriptional responses and a region including this sequence (the δ-domain) is deleted in the oncogenic version of the protein, v-Jun, which does not bind to JNK [54].

The JNK phosphorylation site we identified in MS1 at Thr-62 also lies just downstream of a putative MAPK docking motif (residues 19–27: KVRRATLVI; Fig. 5C). These residues fit the consensus sequence for a so-called a D-domain ([R/K]_2–3_ – X_2–6_ – Ф_A_ – X – Ф_B_) that mediates interactions between MAPKs and their substrates and upstream activators [53]. We were also able to show that JNK and MS1 co-immunoprecipitate (Fig. 4C), suggesting that they interact, either directly or indirectly, in cells. Taken together, these data suggest that JNK may interact with the N-terminus of MS1 and phosphorylate the protein in an analogous fashion to c-Jun.

MS1 is generally thought to be a cytoplasmic protein localised at the myofibril by well-characterised actin binding domains at its C-terminus [1,8]. However, MS1 has also been observed in the nucleus [2,42]. More recent work also provides evidence for a possible Nuclear Localisation Signal (NLS; residues 153–180) and a putative DNA binding domain towards the C-terminus (residues 270–375) which contains an AT-hook motif [42].

The presence of a DNA binding domain, a putative MAPK docking site and a MAPK phosphorylation site within MS1 suggests that MS1 may function as a transcription factor regulated by MAPK signalling pathways to regulate gene expression in muscle cells. Further work using phosphorylation site mutants of MS1 coupled with more in-depth MS1 transcription reporter assays will be required to test if JNK targets MS1 in the nucleus to regulate its transcriptional activity and hence hypertrophic gene expression in response to cardiac myocyte cell stress.

In summary, our results identify MS1 as a transcriptional target for the JNK pathway in cardiac myocytes subjected to metabolic stress. We further demonstrate the physical interaction of JNK and MS1, possibly mediated by a putative MAPK docking site in MS1, and the identification of MS1 as an *in vitro* substrate for JNK with a major phosphorylation site at Thr-62.
